# The future of rare disease drug development: the rare disease cures accelerator data analytics platform (RDCA-DAP)

**DOI:** 10.1007/s10928-023-09859-7

**Published:** 2023-05-02

**Authors:** Jeffrey S. Barrett, Alexandre Betourne, Ramona L. Walls, Kara Lasater, Scott Russell, Amanda Borens, Shlok Rohatagi, Will Roddy

**Affiliations:** 1 Aridhia Digital Research Environment, Glasgow, UK; 2 Critical Path Institute, Tucson, AZ USA; 3 Aridhia Bioinformatics, 163 Bath Street, Glasgow, G2 4SQ United Kingdom

**Keywords:** Rare diseases, Data analytics, Drug development, Digital research environment (DRE), MIDD

## Abstract

Rare disease drug development is wrought with challenges not the least of which is access to the limited data currently available throughout the rare disease ecosystem where sharing of the available data is not guaranteed. Most pharmaceutical sponsors seeking to develop agents to treat rare diseases will initiate data landscaping efforts to identify various data sources that might be informative with respect to disease prevalence, patient selection and identification, disease progression and any data projecting likelihood of patient response to therapy including any genetic data. Such data are often difficult to come by for highly prevalent, mainstream disease populations let alone for the 8000 rare disease that make up the pooled patient population of rare disease patients. The future of rare disease drug development will hopefully rely on increased data sharing and collaboration among the entire rare disease ecosystem. One path to achieving this outcome has been the development of the rare disease cures accelerator, data analytics platform (RDCA-DAP) funded by the US FDA and operationalized by the Critical Path Institute. FDA intentions were clearly focused on improving the quality of rare disease regulatory applications by sponsors seeking to develop treatment options for various rare disease populations. As this initiative moves into its second year of operations it is envisioned that the increased connectivity to new and diverse data streams and tools will result in solutions that benefit the entire rare disease ecosystem and that the platform becomes a Collaboratory for engagement of this ecosystem that also includes patients and caregivers.

## Introduction

### Rare disease drug development: current challenges and opportunities

Current estimates suggest in excess of 395 million of the 7 billion humans worldwide live with a rare disease. Although rare disease may be rare, rare diseases are not. Despite the name and diverse disease phenotypes, rare diseases collectively affect a huge number of people—approximately 3.5–5.9% of the world’s population, according to a recent estimate [1]. Moreover, this global community and collective target population is acutely affected by issues that are systemic to general biomedical research.

**Fig. 1:**
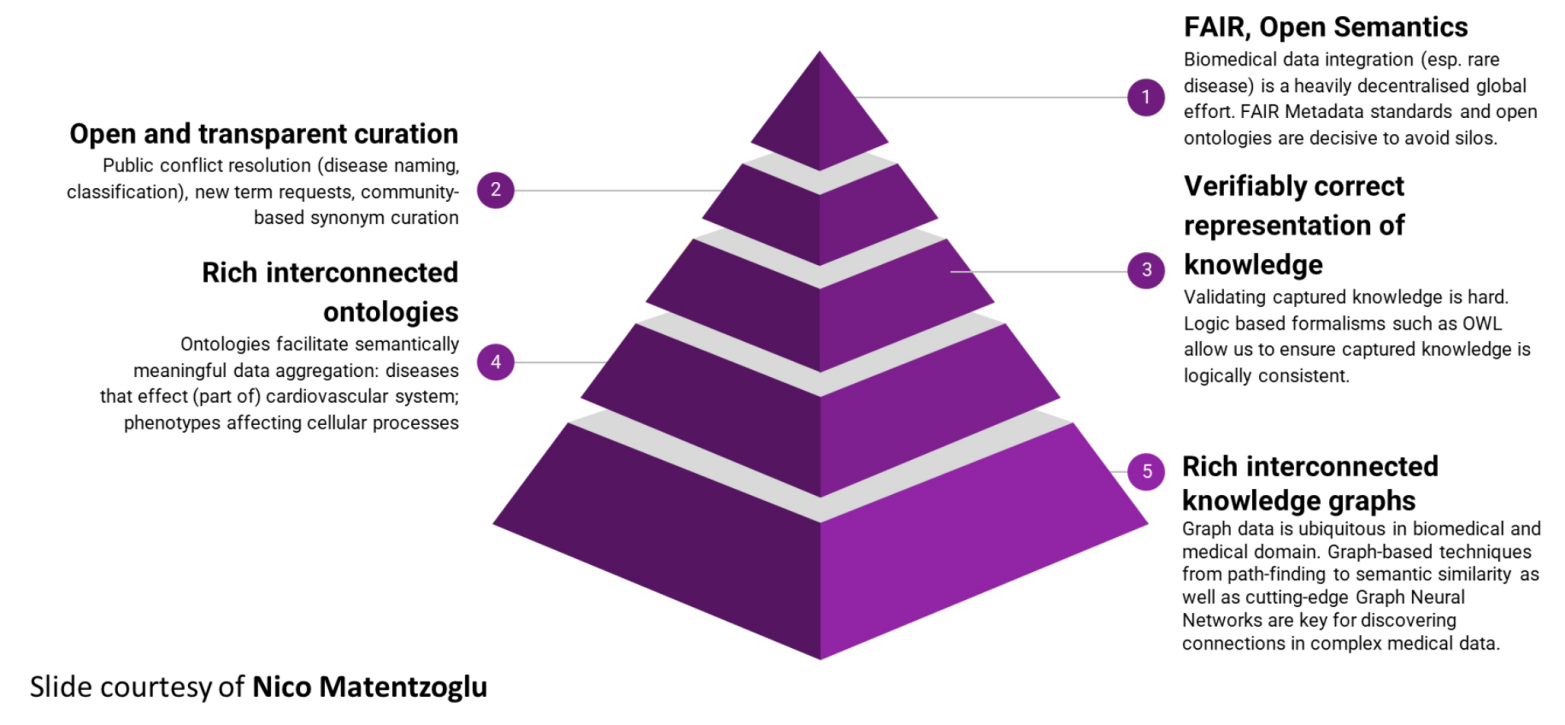
Maslow Pyramid of Knowledge Needs for biomedical data integration *Figure courtesy of Nico Matentzoglu*

Global legislation has provided incentives to drug developers that made it more attractive to investigate therapeutics for rare diseases [2–4]. Without such legislation there would be less progress than we have seen to date in fighting rare diseases. Since the Orphan Drug Act was passed there have been 500 drugs approved in the United States for rare diseases [2]. It’s unlikely that there would have been that many without the legislative incentives. Rare and orphan diseases represent a significant public health priority and an area of great unmet need. Development of therapies for these diseases has been slow, due in part to limited biological understanding of most conditions, challenges in running clinical trials in small populations, and small market sizes resulting in limited cost return on therapies [5, 6], once their efficacy is demonstrated in clinical trials. Many of the challenges to rare disease drug development are captured in Table 1. The Rare Disease Cures Accelerator – Data and Analytics Platform (RDCA-DAP) was established as a partnership between the Critical Path Institute (C-Path) and the National Organization for Rare Disorders (NORD). RDCA-DAP aims to improve the quality and the accessibility of rare disease data, centralize existing datasets in aggregate so that new studies may be informed by the totality of data available, improve the quantitative understanding of rare disease natural history, and help researchers leverage and analyse data to inform and optimize clinical trial design. The platform will support the use of data to define novel biomarkers and endpoints and provide analytical tools to help inform the design of innovative trial protocols that advance rare disease therapy development. In short, much of the added value for collecting this target data within RDCA-DAP is the integration of the diverse data types to create useable data (see Fig. 1) so that more tangible solutions for patients can ultimately be attained.

**Table 1:** Current Challenges for Rare Disease Drug Development

Challenge	Impact
Enrolling, engaging and retaining patients	Delays time to conduct, complete and report on clinical trial results – delays submissions
Designing and evaluating clinical trials	Creates uncertainty with trial outcomes and confidence among regulators that results are interpretable and generalizable
Ensuring the quality of patient data	Creates uncertainty with trial outcomes and confidence among regulators that results are interpretable and generalizable
Global regulatory requirements and payer evidence	Creates uncertainty among payers and patients that results will improve meaningful patient outcomes, QOL and ultimately reduce healthcare costs
Poor understanding of the disease process and natural history	Poorly designed trials, inappropriate biomarkers and endpoints or poor patient selection
Incomplete understanding of clinically-meaningful endpoints	Uncertainty with trial outcomes that results are interpretable and generalizable
Inability to assess clinical benefit and achieve full approval	Uncertainty among payers and patients that results improve meaningful patient outcomes, QOL and ultimately reduce healthcare costs

From a drug development perspective, the primary challenge to this market remains the balance of funding R&D while market opportunities on the commercialization end remain constrained by small patient populations and likewise market size [7]. Financial incentives for rare drug development in the US were codified in the 1983 Orphan Drug Act (ODA) as stated above. These incentives include tax credits, waivers of US Food and Drug Administration (FDA) user fees, and 5 years of marketing exclusivity for rare indications. Numerous programs now exist in the US to de-risk drug development. Prominent among these are NIH National Center for Accelerating Translational Science’s (NCATS’) Therapeutics for Rare and Neglected Diseases and Bridging Interventional Development Gaps programs which provide access to NCATS expertise and contract resources to conduct crucial preclinical studies necessary for regulatory approval of first-in-human trials. Other initiatives seek to incentivize rare disease drug development, including voucher programs (e.g., for rare pediatric diseases), grant programs (e.g., enabled under the Rare Disease Act of 2002), Small Business Innovation grants/contracts, targeted research efforts (e.g., Rare Cancer Moonshot) and others as well as regulatory pathways (e.g., Accelerated Approval). Outside of the US, incentives for development, as well as patient access to resulting treatments, vary widely by country and region. A key question for PhRMA (Pharmaceutical Research and Manufacturers Association) is whether this is sustainable and how it compares to other therapeutic areas that compete for internal and external resources as they attempt to manage and refine their portfolios.

An interesting sidebar in the drug development in general but especially rare disease drug development is the issue of data relevance and the usual landscaping effort that precedes project team engagement and the development of the initial target product profile (TPP) draft. Part of the challenge in this initial exercise is the backdrop of data silos that have historically defined the rare disease ecosystem (pre-RDCA-DAP) and the lack of incentives that have defined the poor data sharing practices. Navigating this situation had been a problem for sponsors or anyone seeking to make use of available data. When coupled with the fact that digitization and enrichment of data through descriptive metadata was the anomaly and not the standard, it was difficult to make use of any data you could find.

One of the important aspects of data collection in general and certainly for data sharing is the notion of more modern data services. Over time, the concept of making data available and useable by the research community has gained traction, and the recognition of the value of sharing data continues to grow [8]. The principles of “FAIR” data, or data that are Findable, Accessible, Interoperable and Reusable, have been widely recognized as an ideal [9]. Data sharing practices, however, remain limited due to concerns around patient privacy, limited data collection, perceived value of individual datasets, reliance on data assets and collaborations as a source of sustainable organizational revenue, and other concerns (see supplementary material). However, shared data that can be made interoperable and reused in combination with other data types and sources are essential to high quality and effective rare disease drug development. In the context of rare disease drug development, FAIR data that has highest value and reduces time to impact. Of course, FAIR data services are only one aspect of operationalizing data into a useable format from which disparate data can be standardized and made available for integration as described in Fig. 1.

**Fig. 2:**
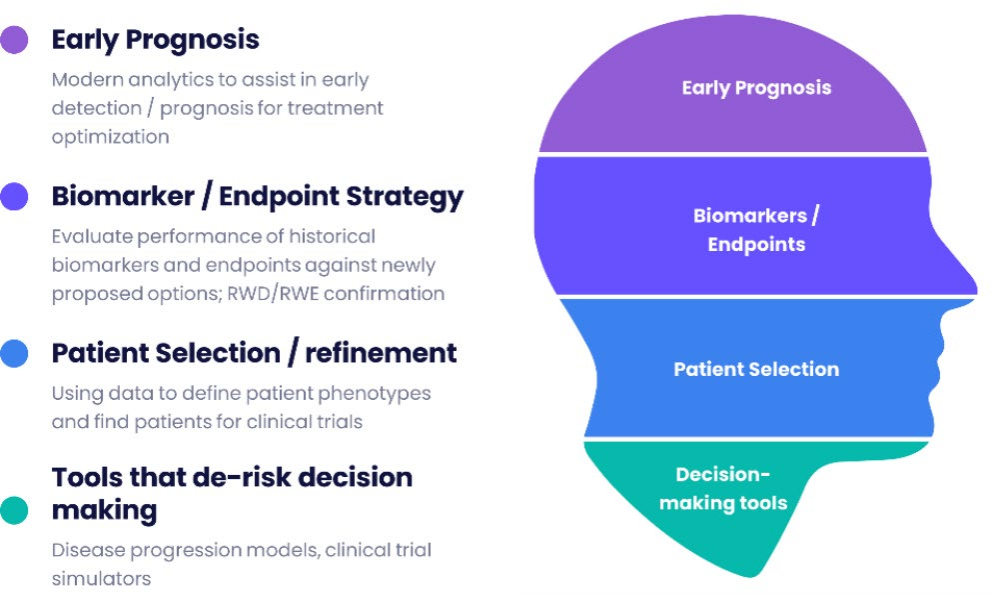
Initial concept space for RDCA-DAP facilitated solutions to accelerate rare disease drug development

This manuscript provides an update on the creation, current implementation and future development of the rare-disease data analytics platform (RDCA-DAP), describes the mechanics of the digital research environment (DRE) that underpins and powers the platform and the mechanism by which the platform is operationalized by the Critical Path Institute (CPATH). It describes the pathway by which the platform has evolved to become a Collaboratory for rare disease drug development including the generation of actual solutions that facilitate rare disease drug development. We define a solution in this context as being more closely linked with actual assets to accelerate an approval. Figure 2 provides a broad categorization of solutions for rare disease drug development all of which we believe can be facilitated with the RDCA-DAP platform.

### Data relevance to collaborative research and rare disease drug development

Conventional drug development proceeds in a somewhat sequential manner in which series of well-defined preclinical and clinical experiments are planned, designed and conducted with the data describing these experiments, being formatted, structured and compliant with regulatory standards and expectations. More and more and especially for rare disease drug development additional data, often not generated by the sponsor are used to augment decision making and are often incorporated into the final regulatory submissions. These data can include various real word data (RWD) sources such as natural history data [10], electronic medical records [11] and data from patient-centric registries [12]. Such data are often unstructured, may be unformatted and not compliant with current data standards and likewise require curation and other means to ensure that they are interoperable or otherwise suitable for integration with more structure data.

There are concerns that the current research and development landscape sets many projects up for unnecessary failure, particularly in the rare disease ecosystem, and does a disservice to rare disease patients [13]. This encourages the collection of redundant data in uncoordinated parallel studies and registries to ultimately delay or deny potential treatments for ostensibly tractable diseases; it also promotes the waste of precious time, energy, and resources. Groups at the National Institutes of Health and Food and Drug Administration have started programs to address these issues. This was also the impetus behind the funding provided by FDA for the creation of the RDCA-DAP in the first place as data quality and integration were viewed as current bottlenecks in accordance with other issues listed in Table 1.

### Voice of the patient

Some of the inherent and practical challenges to conducting clinical trials and selecting or developing endpoints for rare disease clinical investigation also benefit by including the patient voice or perspective. Challenges include the lack of regulatory precedent for some proposed endpoints, a void of available measures, little or no published literature or natural history information, the practicalities of obtaining access to patients, and the appropriateness of placebo-controlled trials [5]. The patient perspective is a critical component in both defining treatment benefit and in interpreting the meaningfulness of a change (or lack thereof). Engaging with patients is needed at multiple steps along the long road of drug discovery and development. They are clearly a vital part of the rare disease ecosystem and likewise sure be a part of the community that contributes and discusses data and various analyses. Likewise, advances in genomic technologies have facilitated novel breakthrough therapies, whose global developments, regulatory approvals, and confined governmental subsidisations have stimulated renewed hope amongst rare disease patient organisations (RDPOs). With intensifying optimism characterising the therapeutic landscape, researcher-advocate partnerships have reached an inflection point, at which stakeholders may evaluate their achievements and formulate frameworks for future refinement [14].

A recent trend is in patient groups, or in some cases individual patient advocates, seeking to create their own collaborations, funds, and research networks to address rare diseases. In some cases, these patient-led models are blending “traditional” venture-backed biotech approaches with philanthropic funding, cooperatives, and other models to create new and innovative means to accelerate discovery and approval, simultaneously seeking to prioritize the patient perspective. Other patient-led innovations include networks for data sharing and analysis, including RARE-X, NORD IAMRARE, and Genetic Alliance PEER, that enable patients to share personal health data with researchers and industry.

RDCA-DAP was launched to break down barriers among rare diseases data silos, and establishing an integrated platform able to accommodate the multiple sources of patient-level data regardless of its source. Patient-powered registries and research networks developed by patient organizations as described above are rapidly evolving leading to significant improvements in patient engagement, in research, care, and health [15]. These patient-powered registries are creating tools and resources to provide more sophisticated ways to tailor patient group engagement in the research process, but few of these engage patients to work with drug development scientists to develop decision-making tools in any meaningful way. Likewise, there is little interaction or feedback from drug development scientists and patients. The notion of including patient voices in the construction of decision-making tools represents a tremendous opportunity in developing therapies tailored to improving patient needs or quality of life (QOL) indices, guided by the “voice of the patient”, especially in diseases where there are insufficient biomarkers or endpoints to pivot development towards.

## Platform concept

### Value of information and more than information

Information value and weight of evidence are often used interchangeably and relate to the notion that having lots of data (amount unspecified in this context) does not guarantee one utility for knowledge or prediction. The origins of the terms are often linked to the financial sector though the subject matter and intention goes beyond that popularization [16]. The weight of evidence tells the predictive power of an independent variable in relation to the dependent variable. Since it evolved from credit scoring world, it is generally described as a measure of the separation of good and bad customers. “Bad customers” refers to the customers who defaulted on a loan and “good customers” refers to the customers who paid back loan. In the context of rare disease drug development, information value needs to be examined in the context of usefulness for the development of actual solutions that accelerate drug development. Utility then can be quantified based on the importance of data to support an assumption regarding a key component of a model or tool for example. Likewise, additional data should add some incremental benefit or increase confidence in the assumption as opposed to redundant information that is either non-informative for a specific purpose or altogether adds no incremental benefit. Consider the key decision criteria of patient selection, dosing, biomarker or endpoint selection as drug development milestones which are hopefully informed by data.

Improving data quality is also seen as an essential element of the platform with the intention of improving the value of the information. Again, with respect to the operationalizing of source data ingestion, curation and integration we are still somewhat in the evolutionary phase relative to the speed and efficiency we desire in the future. A few aspects should be called out as central to advancing the desired data services. These include but are not limited to the following: ingestion diversity beginning with ‘simple’ data types and minimal curation to more complex curation requirements, moving to enriching data, adding more value to the raw data through descriptive field-level metadata, data typing, ontology information, and standard formats such as SDTM, building a body of data and knowledge that can be catalogued and made searchable in one place leading to additional value from the ecosystem and pooled knowledge including standardization and harmonization of data from different sources and finally moving to different data types and learning how to bring these together coherently (e.g., how to index structured and unstructured data together and make them searchable and meaningful to researchers in the same place – example of tabular data vs. medical imaging data). Additionally, exploring ways to identify connections across different types of data using common ontological concepts thus maximising impact of the limited selection of available rare disease data is clearly among the future expectations surrounding data handling and the implementation of FAIR principles. For example, since individuals may have multiple diseases, each with its own diagnosis, the “age at diagnosis” must somehow relate to the disease which was diagnosed at that age.

### A platform for collaboration that engages the RD Ecosystem

Despite the provisions of the Orphan Drug Act, rare disease therapeutic development remains a challenge, especially for very rare disorders [5]. Many of these conditions are still understudied, and even when the genetic cause of the disease is known, understanding disease characterization, progression and clinical manifestation, heterogeneity in the population, and/or how to measure clinically meaningful change due to prospective therapies remains limited. By the very nature of small populations, gathering sufficient data to inform research and provide answers to these unmet needs is challenging, as is running classically designed clinical trials with large enough sample sizes to be better statistically and clinically meaningful. Adding to these complexities, many rare diseases affect geographically and globally dispersed populations which makes identification of patients and longitudinal follow-up challenging. Individuals may show additional variance in disease natural history due to different health systems and different standards of care.

FDA initiated a program called the “Rare Disease Cures Accelerator” to work with the rare disease community to address these issues [17]. This initiative includes the development of a data and analytics platform (DAP), the design of acceptable clinical outcome assessments, and the development of a global clinical trials network. As such, the Rare Disease Cures Accelerator – Data and Analytics Platform (RDCA-DAP) was established as a partnership between the Critical Path Institute (C-Path) and the National Organization for Rare Disorders (NORD). RDCA-DAP aims to improve the quality and the accessibility of rare disease data, centralize existing datasets in aggregate so that new studies may be informed by the totality of data available, improve the quantitative understanding of rare disease natural history, and help researchers leverage and analyze data to inform and optimize clinical trial design. The platform will support the use of data to define novel biomarkers and endpoints and provide analytical tools to help inform the design of innovative trial protocols that advance rare disease therapy development. While working with existing data to improve therapy development now, RDCA-DAP is also working towards establishing standards for regulatory-ready natural history data collection over time, to further improve the rare disease drug development ecosystem.

It is hoped that RDCA-DAP can serve as a model for patient-focused drug development incorporating the voice of the patient but managing both regulatory interest and PhRMA engagement. The role of patients and parents has been clearly articulated by others [18, 19] and the evolution of the platform is clearly planned on additional and customized engagement with patients and parents.

## Platform implementation

### Platform design: role of DRE

**Fig. 3:**
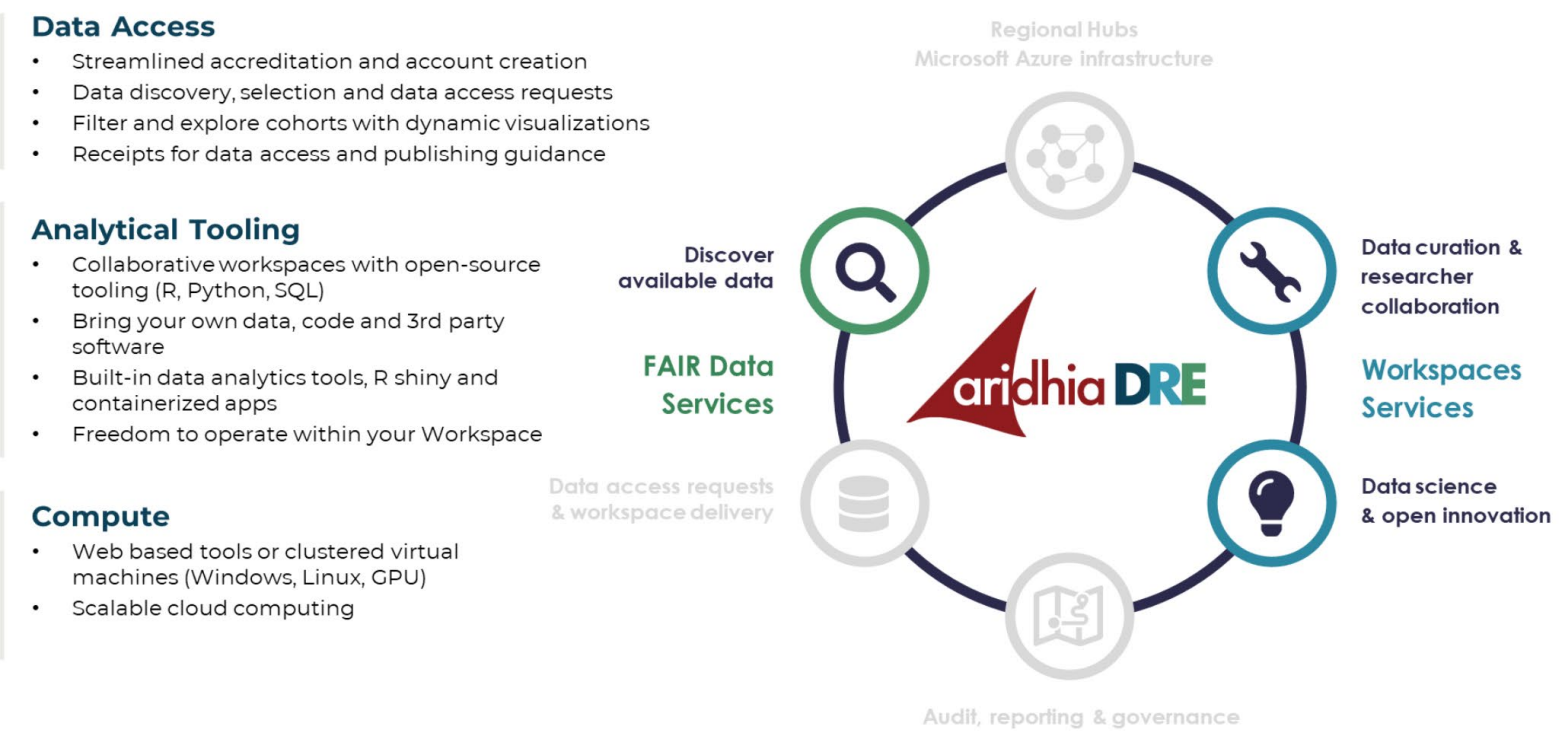
Aridhia DRE Workspace Services

The RDCA-DAP platform is an example of a digital research environment which can also be described as a trusted research environment (TRE). In general, a TRE is a secure collaborative research environment for digital analysis of data. TREs can only be accessed by approved researchers. Governance processes and TRE features ensure only approved researchers can access data, and no data enters or leaves the environment without the express permission of an approved proxy for the data owner. Because data stays put, the risk of patient confidentiality is reduced. Essential companions to the TRE are dynamically-updated and searchable metadata catalogs, in situ analysis tools with code versioning, as well as data provenance, and audit trails. Without these, a TRE is simply a safe place for a single project to be completed and then archived with limited usefulness for new projects or data consumers on other project teams. A DRE Workspace is a Trusted Research Environment – providing a safe-haven for clinical researchers, bioinformaticians and pharmacologists to analyze and develop models on sensitive data with the confidence that the data and models developed are secure and protected. A DRE builds on the concept of a TRE in that it provides remote access to data alongside tools for analysis in a securely controlled workspace, but it also adds essential components that allow the data and tools to be FAIR, version-controlled and dynamically growing in size or quality as a result of each collaboration, and to break down the silos often created by aggregating and analyzing data as a single-use asset. Figure 3 provides a schematic for the various workspace services maintained by the Aridhia DRE that supports the RDCA-DAP platform.

RDCA-DAP integrates existing datasets from various sources within the rare disease community including data from clinical trials, patient registries, preclinical data, natural history studies and electronic health records of individual hospitals and health systems. RDCA-DAP also plans to federate with existing data aggregators and databases via shared data models and application programming interfaces (APIs) to help create an ecosystem of rare disease data and information. The C-Path team have years of experience curating, integrating, and analyzing data and are familiar with common data quality issues such as lack of standardization, missing values, and poor or incomplete data dictionaries. Most of these issues are not unique to the rare diseases data ecosystem but rather widespread issues common to all patient-level data. Issues in quality, completeness, and relevance of source data inevitably lead to uncertainty in downstream findings. When these data are used in applications for regulatory decision making, uncertainties that cannot be addressed through available evidence will translate into gaps in knowledge that should be addressed in the design and conduct of clinical trials for the intended indication. If not, these uncertainties might result in request for additional studies where generating adequate clinical efficacy and safety data may not be feasible. Methodological issues of particular significance arise when using data from sources other than clinical trials conducted according to Good Clinical Practice, or when integrating evidence from different data sources.

### Development history – operationalizing the DRE

**Fig. 4:**
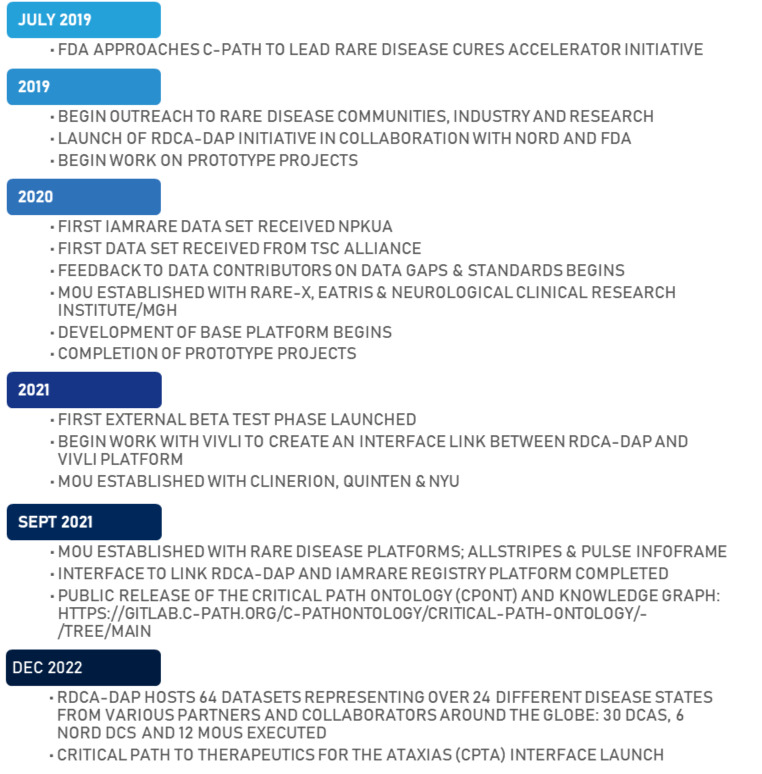
RDCA-DAP development timeline

A timeline for the development, beta-testing, platform launch and post-launch development for RDCA-DAP is shown in Fig. 4. Prior to the public launch of RDCA-DAP, it was important to seek input from platform users and stakeholders that were external to the development. A subset of stakeholders participated in a beta to test and evaluate the platform and provided valuable feedback about RDCA-DAP features, usability, performance, and workflows.

The beta tester roster was developed with input from RDCA and C-Path leadership to include individuals from five main stakeholder groups: academic, quantitative modelling, industry, regulatory and patient registries/groups. Thirty-four potential testers were identified, with a goal of having one to two individuals from each stakeholder segment. The final group included eleven beta testers who were given access to FAIR Data Services and analytical Workspaces.

The RDCA-DAP beta ran from April to May 2021 and resulted in 12 beta workspaces, 8 active beta testers, and a 75% post-beta survey response rate that provided key feedback for improvements prior to public launch. Beta testers utilized platform resources such as dedicated learning courses, a Knowledge Base, and automated sign-up and tooling workflows to get started. From feedback collected during and after the beta, improvements were identified for platform intuitiveness, tools for working with data, and deploying ready-made analytical environments. Further, it highlighted the value of adding a new platform feature to preview and select subsets of data, or cohorts, of interest and improve built-in tools for analyzing large datasets. These improvements were investigated and developed throughout 2021 and into 2022 as improvements deployed every two weeks to the live platform.

On September 2021, RDCA-DAP was publicly launched with improvements to guide users with a dedicated RDCA-DAP information portal, an integrated data access request workflow process, fully equipped researcher workspaces, and improvements to usability and platform integrations. Through the course of a successful annual workshop, new interest and engagement generated eight research workspace requests and 111 active platform users.

Following public launch, platform development focused on incorporating ongoing user feedback and input from the rare disease community. Notably, it was recognized that built-in analytics tools, flexible frameworks, and support for a bring-your-own tooling model helped users become productive more quickly. Further expanding platform performance capabilities, ease of use, and processing were additional improvements key to handling large datasets and computing workloads. Engaging with the community for collaboration across stakeholder groups about future uses and interests also highlighted the importance of improving data sharing, curation, and processes for data access.

At the September 2022 annual workshop, stakeholder groups spanning patient advocates, industry, regulatory, and academia gathered to review platform progress, improvements, and discuss its future direction. Feedback that led to improvements with tools for curating and standardizing data, search features tailored to discovering rare disease data, and new data visualization tools for users to explore the available data and visualize interactive data filters for researchers and patient advocates alike. Engaging with in-person and virtual attendees allowed for insightful conversations about continuing efforts to make data sharing and usage more transparent, demonstrate the value and impact of data, and how to continue seeking conversations to educate and advocate for others in the community.

### Governance and stakeholder engagement

RDCA-DAP governance was developed to facilitate data sharing from all types of stakeholders in the rare disease ecosystem, and encourage open-access to patient-level data by external users. It includes processes and documents established to ensure patient privacy, data security, and respect data contributors’ conditions of use of their data on the platform. The processes and agreements in place were refined prior to the public launch of the platform through numerous interactions with industry, registries and patient-advocacy groups. This led to multiple adjustments to provide sufficient flexibility, key to gain the trust of potential data contributors. In particular, a common worry from contributors relates to losing control on who can access their data, which requires flexibility in how we open data access to users. Sharing data to RDCA-DAP is negotiated through a Data Contribution Agreement (the DCA). The DCA contains non-flexible terms, in particular it binds the contributor to providing patient-level data for which they have proper consent to share and sent with the proper level of de-identification. It also authorizes RDCA-DAP to publish the datasets metadata (including data dictionaries), our standard to follow the FAIR principles. Finally, it authorizes us to use the data internally, and share the patient-level data to regulatory bodies, if requested during a regulatory submission. The DCA specifies that ownership of the data is unchanged, RDCA-DAP never own contributors’ data. This is critical as we encourage broad sharing of data, and do not want to impede sharing to other platform or contributors’ collaborations with industry outside our involvement. Finally, the DCA summarizes how the data provided by our contributors may be requested and accessed by users, how the decision on granting access is determined, and what is requested from users before accessing the data. This is where flexibility is imperative to maximize data-sharing, and answer frequents blocks or concerns. Our DCA encourages data contributors to make their datasets available to users after review by our Data Use Committee (DUC). The five-member DUC is comprised of representatives of C-Path, NORD and other interested parties in the rare disease community, including patient advocacy, academic research and industry.

**Fig. 5:**
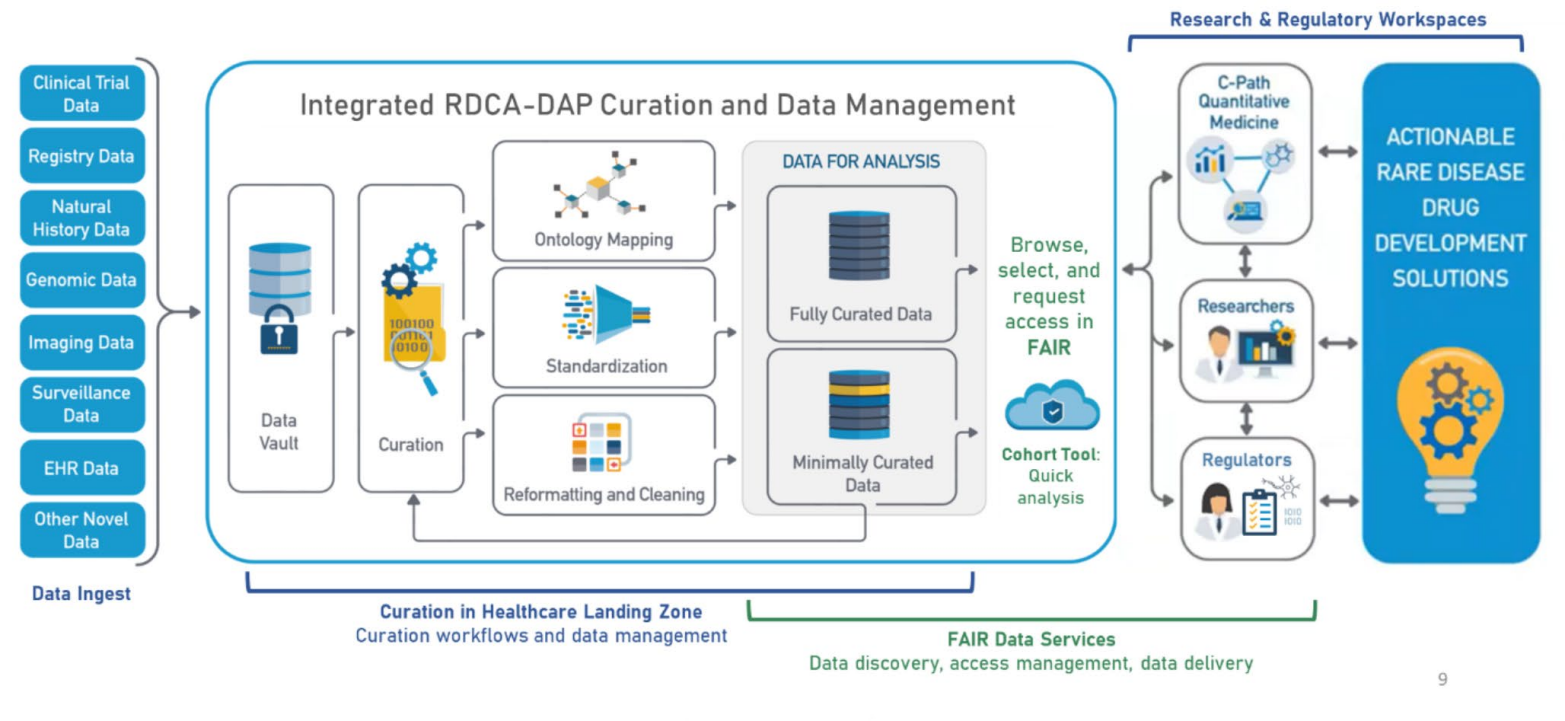
RDCA-DAP Architecture (**A**) and Collaborative cycle and impact on rare disease community (**B**)

In order to request access to patient-level data, the platform users are requested to submit a research project including a project synopsis, publication plans and projected timelines. The committee experts examines users’ requests for access based on the scientific merit of the research project, educational reasons or public interest rationale, and votes as a majority to determine approval or rejections of the users’ applications within two weeks. At the request of contributors, the DCA can be modified to enable three other options. The data can of course be made non-confidential, granting semi-automatic access to the patient level-data. Sometimes, contributors which to keep the data confidential to C-Path, mostly for publication scooping concerns, or other ongoing work or submission that need the data to stay protected from external access. In those instances, we encourage the installment of a time-limited embargo on the data, after which requests will go to the DUC. Finally, we enable contributors to review the users request and send them the users research projects. We acknowledge that in most instances the original contributors are best positioned to evaluate the suitability of their data to answer the users scientific questions. This may also help them develop collaborations through direct interactions with our users. In that instance, contributors have two weeks to send RDCA-DAP their decision. If that delay is not respected, the DUC process takes over to grant or reject access to the dataset. Once access to one or multiple datasets is authorized, users are required to sign the Data Use Agreement (DUA), a document defining the conditions, limitations and obligations for use of the data. The DUA contractually obligates the data user to only use the data for the research described in the approved research plan, to comply with all applicable laws and regulations and forbid any attempts to re-identify study subjects. For more details the DUA is accessible from this location: https://c-path.org/wp-content/uploads/2022/02/RDCA-DAP_DataUseAgreement.pdf. Additional information and access to governance and DUA documents can be found under the “Data Use’ tab at https://c-path.org/programs/rdca-dap/. Figure 5 illustrates how the platform architecture is managed, operationalized and ultimately governed by the Critical Path Institute’s staff with the ecosystem and feedback regarding additional functionality with external solution developers and the Aridhia technical staff.

With the intention of getting feedback on useability, several sessions (TAG, technical advisory group meetings) were hosted on behalf of FDA reviewers. In addition, student internships were funded to, in part, obtain first hand feedback regarding the ease of platform access and analysis potential from those not recognized as quantitative scientists but still knowledgeable form a life science perspective. In general interns found that using the platform was easy, especially requesting permission to access datasets, and due to the strong integration between the FAIR Data Services and Workspaces it also was simple to find new data. The datasets were perceived as large, supporting traditional statistical inference testing with high power though lacking in the number of rare diseases for which this was the case. The only difficulty reported was actual data extraction once permission was granted; observing that the process seemed slightly convoluted. There was recognition that the website providing tutorial videos explaining how to code and how to navigate the website in case of any minor difficulties was useful. Interns also appreciated that the platform provided users with in built statistical tools that could select variables and generate figures and plots along with the R code which proved to be an incredible resource for a multitude of wrangling and code references. Though there was a request for additional prediction engines and languages. Ultimately, as the user community grows, the development team expects to secure additional feedback so that future enhancements can be guided by the user community.

### Future development and enhancements

**Fig. 6:**
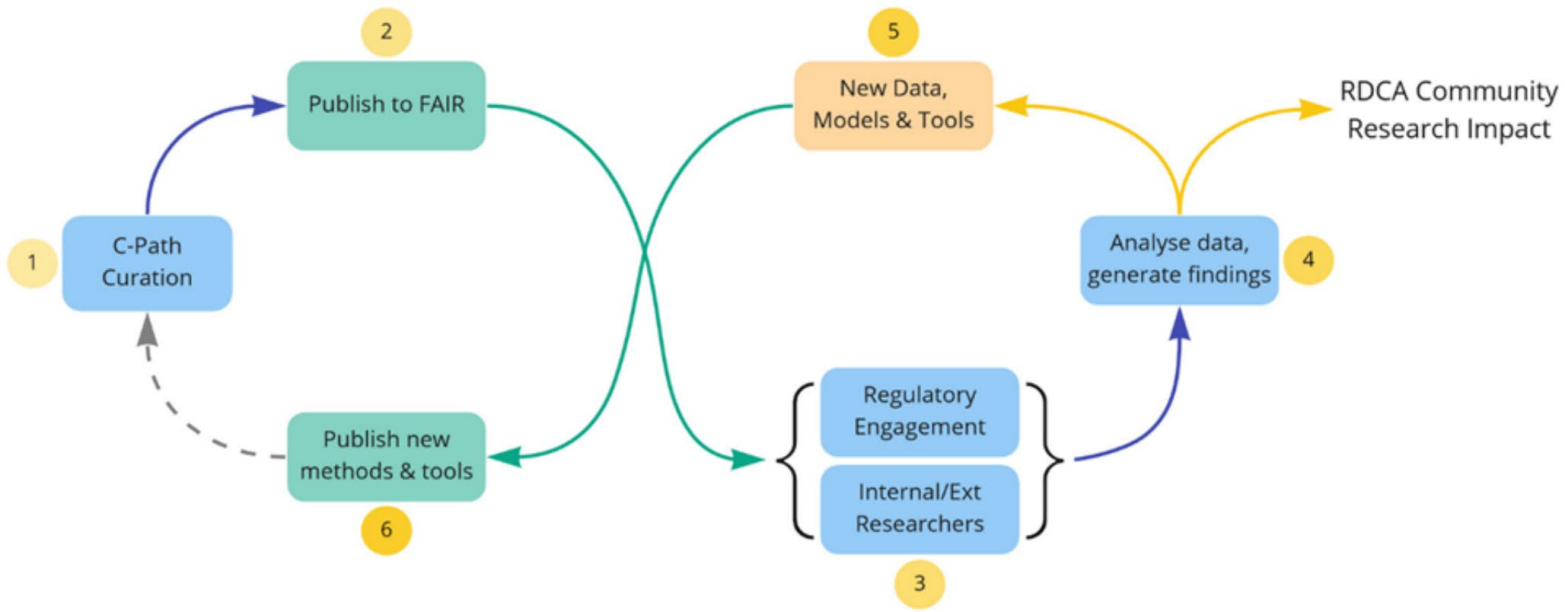
Rich interconnected data (knowledge graphs) to support the generation of RDCA-DAP enabled solutions supporting rare disease drug development

Post RDCA-DAP launch in the fall of 2021, new datasets and features continue to be included in the platform. Short to medium term developments include improved data findability via catalogue (i.e. metadata) customization and enhancement and release of the cohort builder tool to decrease the time to value by understanding if data is suitable for a study and results will be statistically significant. We are also working on semantic search based on a rich interconnected knowledge graph (see Fig. 6) that promotes connections among hitherto unrelated data. The knowledge graph uses the open-source Critical Path Ontology (CPONT) [20] and incorporates both curated knowledge (e.g., anatomical references from upper anatomy ontology UBERON [21]) and individual patient level data from RDCA-DAP. Enhanced governance and data controls will assure contributors that their data are used in accordance with their conditions and provide insight into how data are used. Configurable data access requests will support orchestrated business process management that automates the process of data approval. Long term developments include providing a digital trail of research between connected workspaces, searchable workspaces to build on the outcomes of previous research, and ability for users to request DOIs of datasets and workspaces for digital citation. These enhancements will create the ability to treat a workspace as a digital representation of a research paper, with versioned code, data and outcomes. Finally, configurable data access requests will be pursued to orchestrate business process management workflows automating the process of data approval. It is also envisioned on the short term to encourage collaborative code development. In this manner, development tools and platforms exist within secure workspaces so that code developing models on ‘safe’ data in collaborative teams and airlocking (an isolated and secure environment where collaborators can put their algorithms and models in, execute them against the data, and execute research) to workspaces with robust data security for execution and use by the research team in a secure environment can be encouraged.

Longer term development strategies for the DRE in general include development of a digital trail of research between connected workspaces, searchable workspaces relating prior art and building on the outcomes of previous research (e.g., in a drug development context this could connect key milestones from early stage drug development to activities, plans and outcomes of later stage activities including clinical trials). Enrichment of data, combination of data and analysis, with feedback loop to FAIR is also planned.

**Fig. 7:**
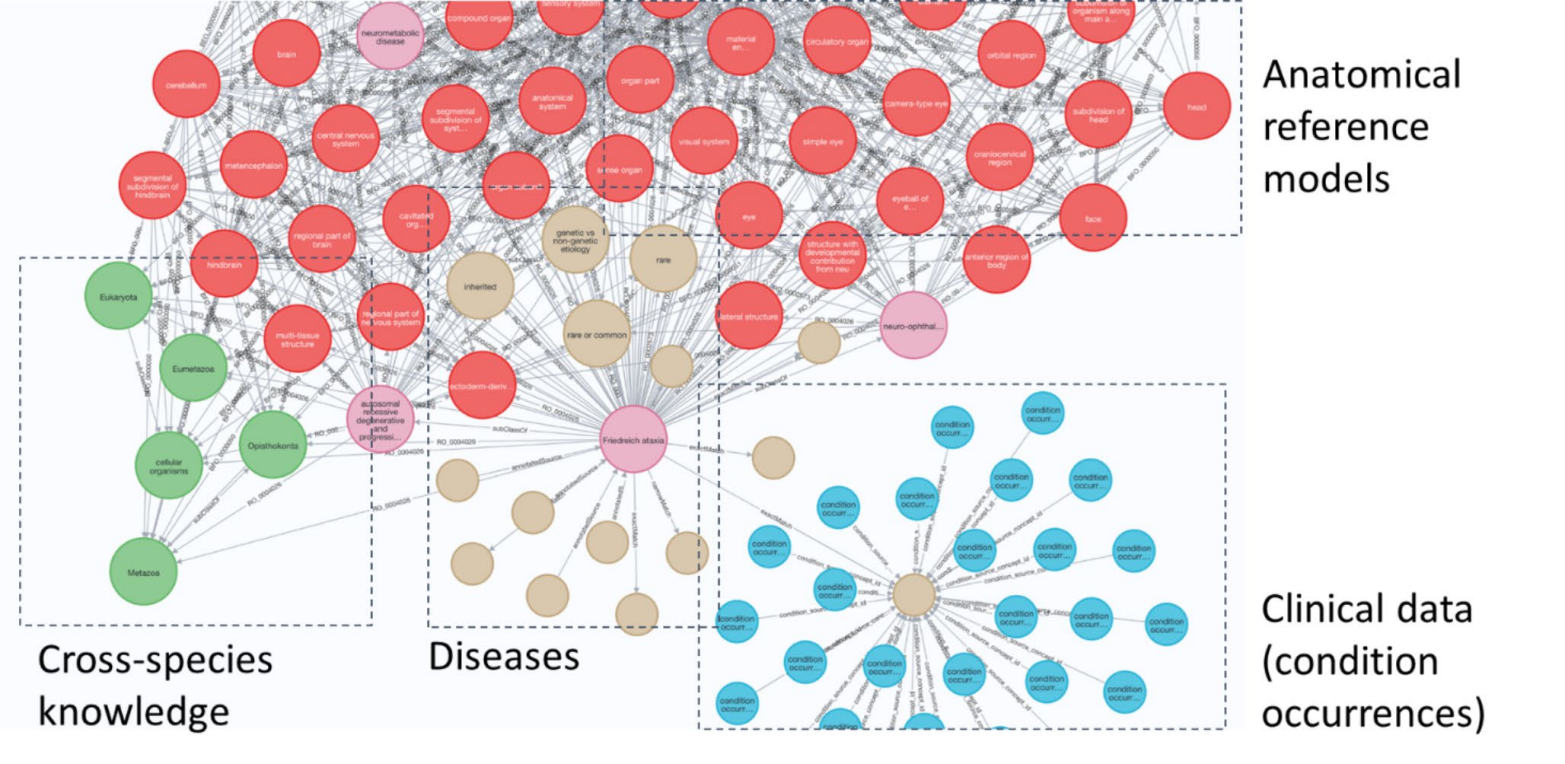
Increased RDCA-DAP workspace functionality example; NLMIX/r workspace integration

The DRE development includes multiple collaborations on workspace functionality already, many of which involve collaborations with open-science developers. These efforts span a diverse group of stakeholders including technology groups supporting early stage drug development (ESQ Labs), those engaged in hospital-based precision dosing initiatives (Great Ormand Street Hospital (GOSH) and Radboud University) and those supporting drug development from discovery through commercialization (e.g., METRUM and Certara). A great hope for this initiative is that the benefit of these enhanced capabilities developed primarily for high income organizations and countries can be shared with others representing low and middle income countries (LMIC) so that the DRE concept can be managed and governed locally by these communities but still allow connection to a global network of researchers seeking to do collaborative research. This would add much value and promote capabilities for all research communities and would make platform trials and similar activities much easier. Early progress on this initiative is very encouraging. Figure 7 provides a screenshot from the open source R-based application nlmixr (https://nlmixr2.org/) implemented within a DRE workspace. While the Aridhia technical staff is working on this with the code developer, efforts within GOSH are being pursued in parallel to not only implement but operationalize this solution for precision doing efforts at their institution. Current and future RDCA-DAP development is thus well aligned with the original objectives provided by FDA but also is responsive to the future intentions for the expanded ecosystem.

## Discussion

Patient-led activism in rare disease financing and discovery is not new, and continues the work led by the National Organization for Rare Disorders (NORD) in the 1980s that resulted in passage of the Orphan Drug Act (ODA) in 1983 and similar legislation globally [22]. ODA provisions include 7-year market exclusivity for orphan drugs, tax credits, development grants, fast-track approval, and waivers of PDUFA fees (a category of FDA user fees for drug developers) all with the purpose of incentivizing orphan drug development. With patient groups, or in some cases individual patient advocates, seeking to create their own collaborations, funds, and research networks to address rare diseases. In some cases, these patient-led models are blending “traditional” venture-backed biotech approaches with philanthropic funding, cooperatives, and other models to create new and innovative means to accelerate discovery and approval, simultaneously seeking to prioritize the patient perspective. The problem with these collective approaches is that they can produce exactly what they are campaigning against – the creation of silos particularly pertaining to data [13]. RDCA-DAP is envisioned as an antidote for such a state.

For RDCA-DAP to fulfil its potential however it must be a vehicle for collaboration and rare disease solutions. Cooperativity between those that construct and improve the functionality of the underlying DRE and those that operationalize the platform to meet the needs of the ecosystem (DCA and DUA agreements, curation, standardization, integration, etc.) needs to be maintained in the presence of a consistent vision for how the platform evolves into a Collaboratory and is sustainable for the future. And additional aspiration is that this truly represents a global solution and is not confined to geographic or political boundaries.

Early progress after more than a year since its launch suggests that the RDCA-DAP platform can in fact be successful at driving solutions that facilitate rare disease drug development [23]. Recently at the World Orphan Drug Conference in Sitges, Spain two case studies were presented that illustrate the promise and the potential [24, 25]. Both represented the development of models / tools that can facilitate the development of rare disease solutions (a clinical trial simulation model for patients with Friedreich’s Ataxia (FA) and a disease progression model for neonates with Bronchopulmonary dysplasia (BPD)). Both works in progress highlight collaborative efforts involving global communities (the Friedreich’s Ataxia Research Alliance (FARA) and the International Neonatal Consortium (INC),respectively) with an invitation to enter RDCA-DAP and further refine the tools. For tools to become solutions however they need rare disease drug developers to use them and incorporate their utility in regulatory submissions that ultimately result in new treatments for patients.

New treatments are not the only goal of the platform and there are several other unforeseen benefits in sight. As maturation of data sciences evolves and ontologies become useful for the discernment of patient phenotype, early patient diagnosis, patient selection for clinical trials, precision medicine and data quality analysis. As those who facilitate the operationalization of the platform engage more with the ecosystem and partner with external collaborators pursuing these approaches the opportunity to develop the science with rare disease data as its use case becomes a reality. RDCA-DAP will obviously benefit from additional data contribution. As data accumulates these can be more appropriately assessed for information value to fill knowledge gaps and eventually an inventory of data by disease type can be generated with the likely requirement of disease data caretakers and additional regulatory input, particularly on the development of solutions derived from the platform with the potential to accelerate rare disease drug development.

While the FDA grant has provided a generous base from which to develop the platform, more diverse funding streams will be required for sustainability of RDCA-DAP as it truly becomes a Collaboratory. The future will hopefully include more data sharing, more solutions and sharing of solutions roadmap’s as well as better usability of all stakeholders and customization of interfaces based on stakeholder type. In the end it will require the community of rare disease stakeholders to provide the necessary input, engagement and investment in RDCA-DAP for the benefit of patients.

## Data Availability

Data generated herein are based on literature and web-review but is available from the PI upon request.
